# Integrating Genetic Counseling in Cardiology Clinics: From Guidelines to Practice

**DOI:** 10.31083/RCM48695

**Published:** 2026-03-10

**Authors:** Despina Sanoudou, Styliani Vakrou, Alexandra Frogoudaki

**Affiliations:** ^1^Clinical Genomics and Pharmacogenomics Unit, 4th Department of Internal Medicine, “Attikon” University Hospital, School of Medicine, National and Kapodistrian University of Athens, 12462 Haidari, Greece; ^2^Cardiogenetics Counseling Clinic, Department of Cardiology, “Attikon” University Hospital, School of Medicine, National and Kapodistrian University of Athens, 12462 Haidari, Greece; ^3^Department of Cardiology, “Attikon” University Hospital, School of Medicine, National and Kapodistrian University of Athens, 12462 Haidari, Greece; ^4^Inherited Cardiomyopathies Clinic, Department of Cardiology, “Attikon” University Hospital, School of Medicine, National and Kapodistrian University of Athens, 12462 Haidari, Greece

## 1. The Role of Genetic Counseling According to the Latest Cardiovascular Disease Guidelines and Clinical Consensus Statements

**Fig. 1.**
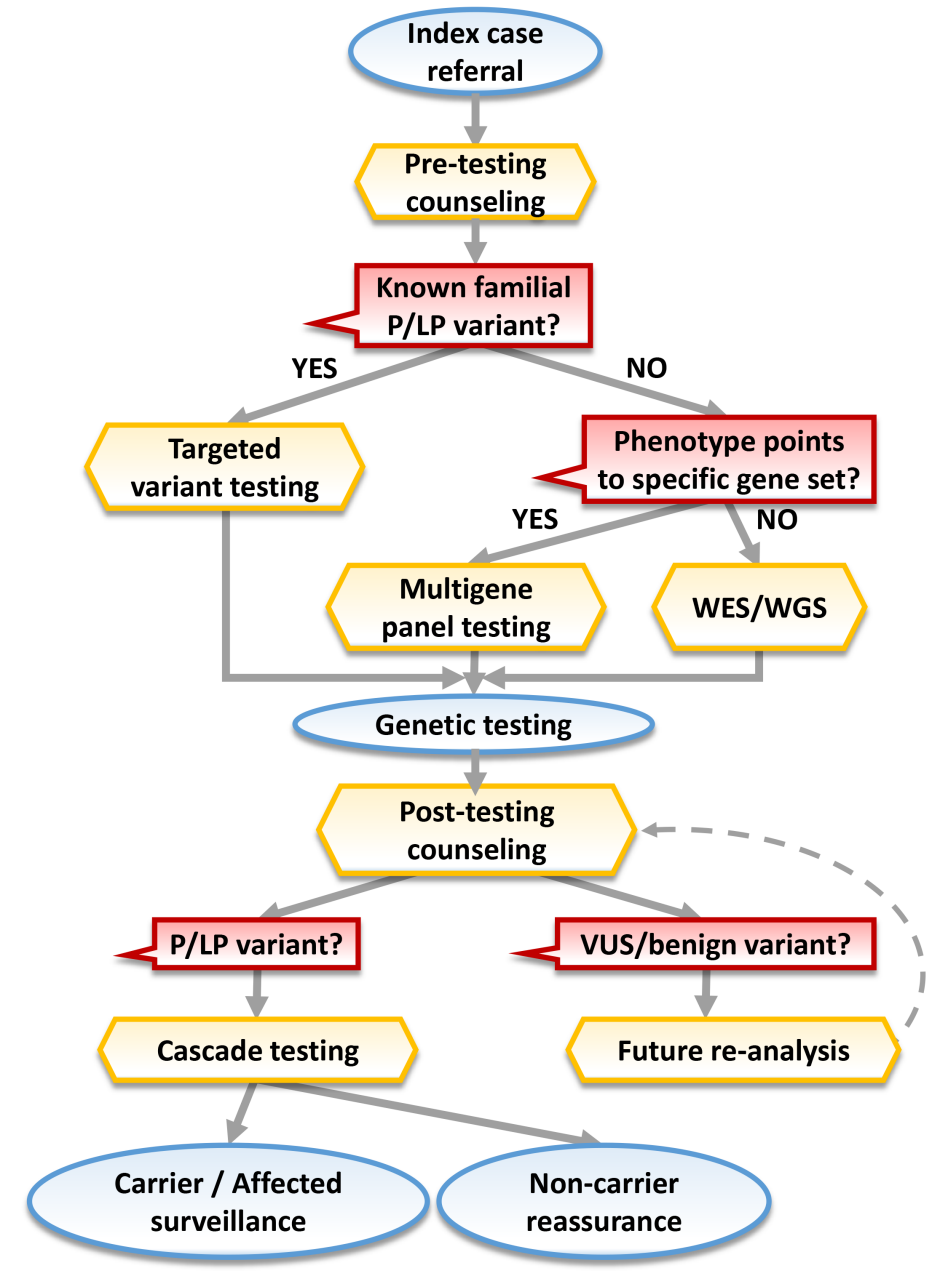
**Flowchart illustrating the integrated pathway of genetic counseling in heritable CVD care**. Yellow: key nodes involving genetic counseling (GC); red: important decision-making steps; blue: clinical management; abbreviations: P, pathogenic; LP, likely pathogenic; VUS, variant of uncertain significance; WES, whole exome sequencing; WGS, whole genome sequencing.

Across the growing body of international guidelines and consensus statements from major cardiology societies, the role of genetic counseling (GC) and genetic counselors has become increasingly well-defined [1, 2, 3, 4, 5]. In recent years, a unified scientific model has emerged in which genetic counseling is designated as a mandatory, structured component of the evaluation and management of inherited cardiovascular conditions, including cardiomyopathies, arrhythmic syndromes, channelopathies, aortopathies, and genetically mediated forms of heart failure. These documents uniformly require pre- and post-test counseling that integrates phenotype-driven test selection, rigorous informed-consent processes, anticipatory guidance regarding the full spectrum of potential molecular findings, and post-analytic interpretation consistent with the American College of Medical Genetics (ACMG)/Association for Molecular Pathology (AMP) standards—particularly the adjudication of variant uncertainty and delineation of genotype-phenotype correlations (Fig. 1). They position counselors as central to coordinated family communication and cascade risk stratification. Together, these documents chart a clear evolution in the field: from recognizing the necessity of genetic counseling to establishing genetic counselors as core clinical partners in the care of inherited cardiovascular disease (CVD) (Table 1) [6, 7, 8, 9, 10].

**Table 1.** **Summary of key recommendations on genetic testing and counseling recently published by major societies**.

Parameter	ESC 2023 Cardiomyopathies [6]	AHA/ACC 2024 HCM [8]	EHRA/HRS/APHRS/LAHRS 2022 [9]
Primary aim of genetic testing	Confirm diagnosis, refine prognosis, inform treatment and reproductive management; enable cascade screening if P/LP variant is identified.	Define etiology and facilitate family management; confirm HCM diagnosis when phenotype is borderline; clarify risk stratification and family counseling.	Establish molecular diagnosis, guide therapy and prevention, enable cascade testing, and contribute to variant interpretation databases.
Pre-test counseling	Mandatory before testing; must include implications for relatives, possible results, and psychosocial/insurance aspects; should occur in a MDT setting.	Strongly recommended; should include discussion of potential outcomes, familial implications, and uncertainty.	Required; informed consent must cover test scope, possible findings, and family implications. MDT including genetic counselor or clinical geneticist is recommended.
Test indications	Offered to patients with confirmed or strongly suspected inherited cardiomyopathy; first-line genes should be *robustly associated* with the phenotype.	Indicated for all patients with a clinical diagnosis of HCM; may be considered for relatives with uncertain phenotype.	Indicated for individuals meeting clinical criteria for inherited cardiac disease or with suggestive family history; broad testing discouraged without phenotype guidance.
Targeted gene panel composition	Phenotype-driven panels focusing on genes with *definitive or strong* evidence; extension to moderate-evidence genes only in research or if clinically justified.	Panels should include *established HCM genes*; broad sequencing acceptable if interpretation expertise and resources available.	Advocates a tiered gene-evidence approach (definitive > strong > moderate); recommends limiting diagnostic panels to validated genes to reduce false positives.
VUS management	*Explicitly stated:* “Cascade testing is not indicated when a variant of uncertain significance is identified.” Segregation analysis may be considered for variant clarification, but not diagnostic.	VUS are not clinically actionable; must not guide therapy or family screening; periodic reinterpretation encouraged.	VUS must be reported separately, with clear communication of uncertainty; no cascade testing; laboratories and clinics must establish re-evaluation pipelines.
Post-test counseling	Post-test counseling mandatory to discuss classification, penetrance, and management; re-classification communicated to patients and families.	Clinician or counselor should explain results, implications for relatives, and need for ongoing surveillance for genotype-positive individuals.	Results delivered with appropriate counseling; emphasis on variant databases (ClinVar, LOVD) and structured data sharing for re-interpretation.
Cascade testing/family screening	Recommended only after identification of a P/LP variant; include pre-test counseling; genotype-negative relatives can be discharged from serial screening; genotype-positive/phenotype-negative relatives require periodic cardiac assessment.	Targeted testing of at-risk first-degree relatives for the familial P/LP variant; negative relatives can be released from surveillance; positive relatives monitored per risk.	Recommended when a P/LP variant is found; requires formal counseling and documented consent; follow local legal/ethical frameworks for minors.
Re-analysis/variant reclassification	Should be implemented at defined intervals (e.g., every 2–3 years) with communication of new interpretations; ideally within an MDT.	Encourages laboratories and clinicians to re-evaluate uncertain or negative results periodically.	Re-evaluation is a shared responsibility of laboratory and clinic; data sharing with registries essential for accurate reclassification.
Service structure/expertise	Genetic testing and counseling should be conducted in a specialized MDT clinic including cardiologists, genetic counselors, and molecular geneticists.	HCM care should involve access to genetics professionals; referral recommended if expertise unavailable.	Testing should occur within a multidisciplinary cardiovascular genetics framework with defined competencies and quality standards.

## 2. The Implications of Genetic Counseling Integration in Inherited Cardiovascular Disease Clinics 

Integrating GC directly into inherited CVD cardiology pathways improves test selection, the clinical interpretation of results, and the identification of at-risk relatives. At the front end, GC-led selection of disease-focused panels - rather than indiscriminate mega-panels, reduces ordering inappropriate tests and the downstream burden of variants of uncertain significance (VUS). Multiple stewardship programs show that review or co-ordering reduces inappropriate or duplicative genetic tests and yields measurable cost savings at the health-system level [11, 12, 13, 14].

Appropriate test selection leads to enhanced clinical utility: in inherited cardiomyopathies and channelopathies, contemporary cohorts consistently report that a meaningful minority of probands harbor clinically explanatory or management-informing variants; approximately two-thirds of positive findings have treatment or surveillance implications (e.g., Implantable Cardioverter-Defibrillator (ICD) timing in LMNA- or FLNC-positive dilated cardiomyopathy (DCM), exercise restriction in arrhythmogenic disease, drug avoidance in channelopathies, and pregnancy counseling). These benefits are better realized when pre- and post-test counseling translate laboratory reports into actionable clinical strategies [1, 15, 16].

Health-economic benefits are described from GC integration in cascade testing, where the counselor coordinates the identification, testing, and follow-up of at-risk relatives. For example, for familial hypercholesterolemia, the U.S. Centers for Disease Control and Prevention classifies cascade screening as a Tier 1 genomic application (implementation-ready). Recent studies show cascade strategies are cost-effective in most jurisdictions, with incremental cost-effectiveness ratios frequently favorable or dominant versus usual care [17]. National guidance (e.g., NICE) similarly endorses DNA-confirmed cascade pathways; success hinges on systematically identifying and engaging relatives [18, 19, 20, 21].

A significant impact is also emerging at the level of patient outcomes. Counselors tailor risk communication to the patient’s level of understanding, cultural background, and family context, ensuring that genomic information translates into comprehension and action. Observational studies in inherited cardiac disease clinics demonstrate greater patient empowerment and more consistent adherence to recommended testing and surveillance [22, 23, 24]. Family communication is best achieved through structured, GC-facilitated tools. For example, a randomized evaluation of a simple booklet (Family Heart Talk) in DCM increased first-degree relative screening with a minimal addition of time spent by clinicians [6, 23, 25].

Furthermore, streamlining of clinical workflows can be enhanced, as counselors educate patients, standardize pre-test documentation (indication, scope, secondary findings, data sharing), harmonize post-test letters with ACMG/AMP terminology, coordinate genetic variant interpretation boards for complex cases, and keep track of important downstream steps [7, 8].

## 3. From Guidance to Practice: The Steep Path to Implementation 

Although GC is predominantly needed in specialized centers for inherited CVD, such centers are not always available or readily accessible. In those settings, it would be meaningful to aim for the integration of GC in non-specialized Cardiology clinics that are likely to be caring for inherited CVD patients. The preferred approach would need to be adjusted to regional practices.

The genetic evaluation of inherited CVD is more complex than the identification of a familial pattern of disease, requiring expert phenotyping and meticulous assessment of the family history to guide test selection, followed by rigorous interpretation of genetic testing results. As a consequence, the implementation of genetic testing has been cited to present challenges involving incorrect or inappropriate testing, errors in analysis, misinterpretations, and problematic VUS follow-up, potentially jeopardizing patient safety [26].

The 2024 EHRA physician survey (357 respondents across 69 countries) assessed real-world integration of genetic testing and counseling for inherited cardiac diseases, and concordance with the 2022 recommendations [9, 27]. It found substantial underuse and variability: 39% reported no or very low annual testing volumes (<10 tests/year), with the absence of a genetic counselor in those centers reported as a top reason for not providing or limiting access to genetic testing, preceded only by the unavailability of a dedicated cardiogenetic service. Across all respondents, regular multidisciplinary evaluations of test results were reported at only a suboptimal rate (42%). Overall, the survey highlights heterogeneous access to GC, uneven infrastructure, and incomplete implementation.

These findings indicate that translating cardiovascular genetics guidance into daily practice remains challenging, despite its transformative potential [28, 29]. Experience across health systems shows that implementation succeeds when organizational readiness and clinical culture progress in parallel [30, 31]. When counseling and testing are commissioned within a unified and standardized pathway, adoption substantially increases [30, 31]. Single health care providers by themselves are unlikely to be able to provide expert care, given the genetic and phenotypic heterogeneity among inherited CVD patients, as well as the rapid pace of genetic knowledge. Ideally, a range of expertise is required, in the form of a multidisciplinary team including clinicians, nurses, geneticists, and counsellors [1]. The National Health Service (NHS) Genomic Medicine Service in England exemplifies this approach: national test directories, coordinated laboratory hubs, and defined standards for consent, reporting, and family evaluation have embedded genomics into the daily practice of cardiovascular care. Conversely, fragmented reimbursement and decentralized delivery, as observed in the United States and parts of Europe, continue to constrain the use of testing despite clear professional consensus [9, 28].

Beyond infrastructure, effective implementation depends on how GC is positioned within the cardiology ecosystem. The evidence now makes it clear that counselors can significantly contribute to the clinical scope and effectiveness by assisting cardiologists to integrate genetic information in clinical decision making, towards refining risk stratification, tailoring clinical management and therapy, as well as guiding prevention across families [9, 23, 25, 32, 33]. When these complementary disciplines converge, genetic testing evolves from a technical procedure into a clinically integrated continuum of care. Highly promising steps towards this direction are being made by leading hospitals in the US and the UK. Most recently, the Cleveland Clinic launched a Cardiovascular Genomics Initiative to integrate genomic evaluation and counseling into routine CVD care [34]. Patient-facing programs have matured at a handful of leading centers, including Mayo Clinic’s Cardiovascular Genomics Program, the Stanford Medicine’s Center for Inherited Cardiovascular Disease, the Massachusetts General Hospital’s Cardiovascular Genetics Program, the Brigham and Women’s Heart & Vascular Genetics Program, the Penn Medicine’s Center for Inherited Cardiovascular Disease, as well as the Royal Brompton & Harefield Inherited Cardiovascular Conditions Service [35, 36, 37, 38, 39, 40].

Lasting progress, however, depends on professional evolution. Cardiologists should acquire functional genomic literacy—not to replace the expertise of geneticists, but to understand when and how molecular data informs patient management [29]. The integration of practical genetics into continuing education is therefore essential. AHA scientific statements support a pragmatic tiered competency approach. Basic-level competencies target safe triage and timely referral: clinicians should (i) recognize clinical and family-history “red flags” for inherited cardiovascular disease, (ii) obtain and document an interpretable multigenerational pedigree and key phenotypic features, (iii) explain at a high level the purpose, potential outcomes (pathogenic/likely pathogenic, variant of uncertain significance, negative), and limitations of genetic testing, and (iv) initiate referral to a cardiovascular genetics service/genetic counselor when specialized test selection, consent, or interpretation is required. Advanced-level competencies support direct management of positive reports within a structured service: clinicians should (i) select the most informative testing strategy (targeted familial variant vs phenotype-driven panel vs exome/genome, including CNV needs) and the optimal proband, (ii) interpret pathogenic/likely pathogenic findings in genotype–phenotype context and translate them into evidence-based management and surveillance recommendations, (iii) manage uncertainty responsibly (including VUS handling, segregation studies, and re-analysis/reclassification workflows), and (iv) operationalize family-based care through cascade screening, communication pathways, and coordinated longitudinal follow-up in partnership with GC expertise [5, 41, 42].

Specialized genetic counselors are increasingly needed, with extended training in CVD [33]. Their training should go beyond general genetics to a cardiology-based competency set. Core domains include cardiac phenotyping proficiency (interpreting electrocardiogram/ambulatory findings, echocardiography/cardiac magnetic resonance, vascular imaging, and lipid phenotypes; recognizing age-dependent penetrance and variable expressivity), CVD-focused test strategy (selecting the most informative proband and genetic test), advanced variant interpretation with explicit cardiology consequences and family-centered cascade care capabilities integrating CVD-specific characteristics (e.g., variable age of onset, variable expressivity, reduced penetrance) [43, 44].

Achieving equitable access remains another measure of maturity. Tele-genetic counseling provides a pragmatic approach for patients in remote or resource-limited settings, expanding reach without compromising quality or ethical standards [45].

## 4. Conclusion 

In conclusion, moving from guidance to practice requires more than resources; it demands a shared vision in which cardiologists and genetic counselors join forces in transforming genomic knowledge into clinical precision and lasting benefit for patients and their families. This pivotal transition will not merely embody technological modernization but a fuller realization of cardiovascular medicine – one that is predictive, preventive, and person-centered.
